# Socio-Cultural and Economic Valuation of Ecosystem Services Provided by Mediterranean Mountain Agroecosystems

**DOI:** 10.1371/journal.pone.0102479

**Published:** 2014-07-18

**Authors:** Alberto Bernués, Tamara Rodríguez-Ortega, Raimon Ripoll-Bosch, Frode Alfnes

**Affiliations:** 1 Department of Animal and Aquacultural Sciences, Norwegian University or Life Sciences, Ås, Norway; 2 Departamento de Tecnología en Producción Animal, Centro de Investigación y Tecnología Agroalimentaria de Aragón, Zaragoza, Spain; 3 Animal Production Systems Group, Wageningen University, Wageningen, The Netherlands; 4 School of Economics and Business, Norwegian University or Life Sciences, Ås, Norway; Institute of Agronomy, University of Lisbon, Portugal

## Abstract

The aim of this work was to elucidate the socio-cultural and economic value of a number of ecosystem services delivered by mountain agroecosystems (mostly grazing systems) in Euro-Mediterranean regions. We combined deliberative (focus groups) and survey-based stated-preference methods (choice modelling) to, first, identify the perceptions of farmers and other citizens on the most important ecosystem services and, second, to value these in economic terms according to the willingness to pay of the local (residents of the study area) and general (region where the study area is located) populations. Cultural services (particularly the aesthetic and recreational values of the landscape), supporting services (biodiversity maintenance) and some regulating services (particularly fire risk prevention) were clearly recognized by both farmers and citizens, with different degrees of importance according to their particular interests and objectives. The prevention of forest fires (≈50% of total willingness to pay) was valued by the general population as a key ecosystem service delivered by these agroecosystems, followed by the production of specific quality products linked to the territory (≈20%), biodiversity (≈20%) and cultural landscapes (≈10%). The value given by local residents to the last two ecosystem services differed considerably (≈10 and 25% for biodiversity and cultural landscape, respectively). The Total Economic Value of mountain agroecosystems was ≈120 € person^−1^ year^−1^, three times the current level of support of agro-environmental policies. By targeting and quantifying the environmental objectives of the European agri-environmental policy and compensating farmers for the public goods they deliver, the so-called “green” subsidies may become true Payments for Ecosystems Services.

## Introduction

Mountains constitute the ecological backbone of Europe, providing essential ecosystem services (ES) [1]. Euro-Mediterranean mountains have a long history of co-evolution with human activities and can be considered as agroecosystems (mostly grazing livestock systems). In many cases, the continuation of traditional farming practices is essential for the maintenance of the biodiversity value [2], the preservation of the cultural landscape and opportunities for recreation [3], or the protection against natural hazards [4]. Mountain agroecosystems are therefore highly multifunctional; in addition to the provision of private goods, such as food and fibre, they also deliver a wide range of public goods [5], [6].

However, in recent decades, the marginalization and abandonment of agriculture has occurred in many European mountain areas and is threatening the delivery of these ES [7], [8]. General socio-economic trends influence this process, which has been driven by a rapid increase in the opportunity costs of labour [9] due to changes in the relative prices of inputs and outputs. Because many outputs (regulating, supporting and cultural services) of mountain agroecosystems constitute non-market goods, farmers have little incentive to provide them. Therefore, public intervention is needed to achieve a desirable level of provision according to societal demands [5], for example with the establishment of Payments for Ecosystem Services.

In Europe, the current debate stresses the need to account for agri-environmental indicators in order to quantify the impacts of agricultural practice on the environment and to shift the emphasis of the Common Agricultural Policy (CAP) toward the supply of environmental goods. Thus, there is a need to objectively evaluate ES and to integrate agri-environmental indicators into policy design.

ES are classified into four groups: provisioning ES (material or energy outputs); regulating ES (biophysical processes providing benefits such as climate regulation or water purification); supporting ES (processes necessary for the production of all the other ES); and cultural ES (recreational, aesthetic, and spiritual benefits). The application of the ES framework to agroecosystems allows for the simultaneous assessment of all goods and services, both provisioning (food products, fibre, *etc.*) and non-provisioning (regulating, supporting and cultural), at the same priority level, as well as the assessment of the trade-offs and synergies between them [10]. Hence, it has the potential to facilitate the incorporation of non-provisioning ES, which mostly constitute public goods, into policy agendas [11], [12], integrating further agricultural and environmental/biodiversity policies.

The ES framework can be embedded in the wider concept of sustainability and likewise involves wide social and economic dimensions. Hence, apart from biophysical quantification, the study of the ES provided by agroecosystems requires the use of other perspectives to reveal these diverse dimensions or values of ES. While there is an increasing body of literature addressing ES linked to mountain agroecosystems in biophysical terms, difficulties to elucidate relationships between concrete farming practices or management regimes and ES delivery remain. Other problems relate to the difficulty to generalized site-specific measurements, the diversity methodologies utilized, and the mismatch of spatial-temporal scales and metrics [10]. Still, biophysical quantification of non-provisioning services is more adequate and precise to measure and monitor the real condition of ecosystems and guarantee their integrity. Socio-cultural valuation enables the quantification of the relevance of ES to people, unravelling dissimilar perceptions between stakeholders with diverse values, interests, experiences and knowledge. Deliberative valuation approaches are praised for uncovering societal motivations for conserving ES [13], allowing for the inclusion of important cultural ES and nonmaterial values in policy design and decision making [14]. Economic valuation (monetization of ES) is highly controversial [13], [15], [16]. Non-material goods are considered incommensurable by many; therefore, economic valuation is assumed to be a driver for the commodification of nature [16] and very difficult to apply for certain (*e.g.*, cultural) ES [17]. Others have a more pragmatic view and defend economic valuation as a tool for change, evidencing how the “economic invisibility” of nature's flows into the economy is a significant contributor to the degradation of ecosystems and loss of biodiversity [18]. Hence, bringing the economic valuation of non-provisioning ES into land use economic decision making can result in substantial benefits to society [11].

However, trade-offs between biophysical, socio-cultural and economic evaluation frameworks have been noted [10]. Methods for measuring value tend to define the values being measured and, as a result of the dominance of biophysical and economic approaches, the values obtained only partially reflect the concerns of the ES beneficiaries and can be biased towards the information provided by markets [19]. Therefore, a combination of disciplines and valuations methods is recommended [15], [16].

The aim of this work was to elucidate the socio-cultural and economic value of a number of ES delivered by a mountain agroecosystems in northeast Spain. We used deliberative methods (focus groups) to identify the perceptions of farmers and other citizens (hereafter citizens) on the most important ES delivered by mountain agroecosystems. We then used survey-based stated-preference methods to rank the ES previously identified and obtain their economic value according to the willingness to pay of the local (residents of the study area) and general (region where the study area is located) populations. A choice experiment was designed for this purpose, and the data were analyzed with a Mixed Logit model. Finally, we derived some implications for agri-environmental policy design in these regions.

## Materials and Methods

The methodological framework follows the recommendations established by de Groot et al. [20]: (i) it is spatially and temporally explicit at scales meaningful for policy design, as both ecological functioning and economic values are context, space and time specific; (ii) it departs from the biophysical quantification of the ES delivered by Mediterranean mountain agroecosystems, to provide solid ecological underpinnings to the valuation of ES; (iii) it is set within the context of contrasting scenarios (policies), recognizing that both the values of ES and the costs of actions can be best measured as a function of the changes between alternative options; (iv) in assessing the trade-offs between alternative uses, the most significant ES for the population are considered, representing different types (bundles) of ES; (v) the societal cost of alternative uses is explicitly incorporated; and (vi) different stakeholders - *i.e.*, the beneficiaries of ES, those who are providing the services and are involved in or affected by the use - are included in the assessment. Finally, a combination of qualitative and quantitative methods, for socio-cultural and economic valuation, respectively, is used.

### The study area

The study took place in the “Sierra y Cañones de Guara” Natural Park (SCGNP), a protected area of 80739 ha in Northeast Spain (47°17′N, 0°13′W). The park constitutes a calcareous mountain range rich in karstic formations with altitude raging between 430 and 2077 m.a.s.l. Precipitation is very irregular, from 900–1000 mm in the northern side of the chain, with some Atlantic influence, to 600–700 mm in the southern, more Mediterranean side. According to the large variation in environmental conditions, vegetation is also very diverse: 49% of the park is covered with shrub rangelands, 29% corresponds to dense forest, 7% corresponds to open forest rangelands, 7% corresponds to agricultural crops, 1% corresponds to mountain summer grasslands and 7% corresponds to unproductive/urban areas. Agricultural land covers 53% of the total area of the park. The main agricultural activity is grazing livestock; the total census in 2000 was 32651 meat sheep, 700 goats, 1199 beef cattle and 259 mares. Grazing areas include private and communal land that is grazed by domestic animals with an average stocking rate of only 0.15 Livestock Units per ha [21]. The agricultural land also includes some permanent crops (mainly olive trees) and cereals.

The SCGNP constitutes a Special Protected Area (EU Birds Directive) that includes three Sites of Community Importance (EU Habitats Directive). Originally created to protect scavengers and other birds of prey, the SCGNP attracts many visitors due to its rich geological (canyons, caves, *etc.*), cultural (prehistoric and megalithic art, traditional buildings, villages) and natural (endangered species, diversity of landscapes, birds of prey and scavengers, *etc.*) heritage.

Four sustainability imbalances related to grazing livestock systems were identified [8] in the SCGNP: low continuity of farming families; intensification of the management system (leading to reduction of the grazing season and fewer grazing animals); degradation of grazing resources (abandonment of remote/marginal rangeland areas); and concentration of grazing in easy-to-work areas. As a consequence, the general process of vegetation encroachment and landscape closure is happening in many areas of the Park [22].

### Socio-cultural valuation and identification of relevant ES

We used qualitative deliberative research methods to measure the cultural values and social preferences in terms of ES and identified those to be included in the economic valuation. We organized five Focus Groups (FG): two with livestock farmers (n = 11) that used pastures within the park, and three with citizens (n = 22) residents in neighbouring cities. To recruit farmers willing to participate in the two FG, we contacted an association of livestock farmers and an agricultural cooperative existing in the area of study. To ensure homogeneous socio-economic profile of the composition of the citizen FG, we organized with one laboratory technicians in a governmental agency for animal health, one with teachers in an institute for secondary education, and one with members of a consumer cooperative. The objective of the FG was to discuss the relationships between mountain agriculture and the environment in the SCGNP. The FG lasted approximately 1.5 hours and were conducted by a moderator according to five general questions. 1. Do you know the term “ecosystem services”? 2. How do you think livestock production affects the environment and vice versa? 3. How do these relationships between livestock production and the environment affect you? 4. What geographical areas/places can you identify that show the effect of livestock on the environment? 5. Should society pay for the delivery of environmental services? Who? In what way? Participants were asked to reflect individually on the questions for approximately 10 minutes before the discussion and to write in their own words some ideas or examples. The sessions were video recorded and transcripts were written for text analysis.

The content of the transcripts and the context and duration of discussions were considered when analyzing the FG data. To summarize and facilitate the presentation of results, we counted the number of times that particular items of information appeared in the texts. The items were classified as provisioning, regulating, supporting and cultural ES, following the classification proposed by The Economics of Ecosystems and Biodiversity [18]. The most important ES within the ES categories identified during the FG were included in the choice experiment detailed below. However, many other items of information relating to the diverse sustainability issues of mountain agriculture were discussed during the FG (see [23] for details).

### Economic valuation of ES

#### Total Economic Value

The contributions of mountain agroecosystems to human well-being have major economic significance [1], although their value is not recognized by markets. All aggregated values provided by a particular ecosystem constitute its Total Economic Value (TEV) [24]. In the socio-cultural valuation exercise described above, four main ES were identified corresponding to the different categories of ES established by TEEB [18]. We presumed that these ES embody the most important use and non-use values of the TEV taxonomy (Table 1; see also File S1 for details on these assumptions). Non-use values are highly based on moral and ethical concerns and therefore can be difficult to estimate; however, choice modelling can be used to assess all the components of TEV [25].

**Table 1:** Attributes, levels (*status quo* underlined) and components of TEV in the choice experiment.

Attribute (ES)	Levels (no. and coding)	ES type and TEV component
Cultural landscape	3: abandonment, current landscape, rich mosaic	Cultural ES. Non-extractive direct use value (recreation)
Biodiversity	3: 7, 11 and 15 pairs of bearded vulture	Supporting ES. Non-use existence value (preservation of biodiversity)
Forest fires	3: 2, 4 and 6 forest fire events per year	Regulating ES. Indirect use value (indirect benefits)
Product quality linked to territory	3: 2, 4 and 6 quality products available	Provisioning ES. Extractive direct use value (food)
Annual cost	5: 15, 30, 45, 60, 75€	

#### Choice experiment design

The public perception and willingness to pay (WTP) for the ES derived from different agricultural policies can be measured using Stated Preference methods designed for valuation of non-market goods [26]. The measurements are obtained using individuals' stated behavior in a hypothetical setting [27], [28]. In particular, we used a survey-based Choice Experiment, where individuals were asked to choose between policy scenarios in a series of choice sets. Each choice set includes three alternative policy scenarios defined by attributes (in our case different ES provided by Mediterranean mountain agroecosystems) and levels of these attributes (Table 1). For example, in our case, one attribute is the conservation status of an endangered species, which can take three levels as a consequence of different policies. All ES attributes have three levels and annual cost have five levels. The definition of attributes and levels is described in detail in File S1. When individuals make their choice, they trade off between the levels of the attributes and the associated costs describing the different policies in the choice set. In the analyses all ES variables are treated as categorical variables, while the annual cost is treated as a continuous variable. Because each attribute (ES) corresponds to a different component of the TEV and all attributes are evaluated simultaneously, the sum of the WTP values obtained in the analysis can be considered the TEV of Mediterranean mountain agroecosystems.

**Figure 1:**
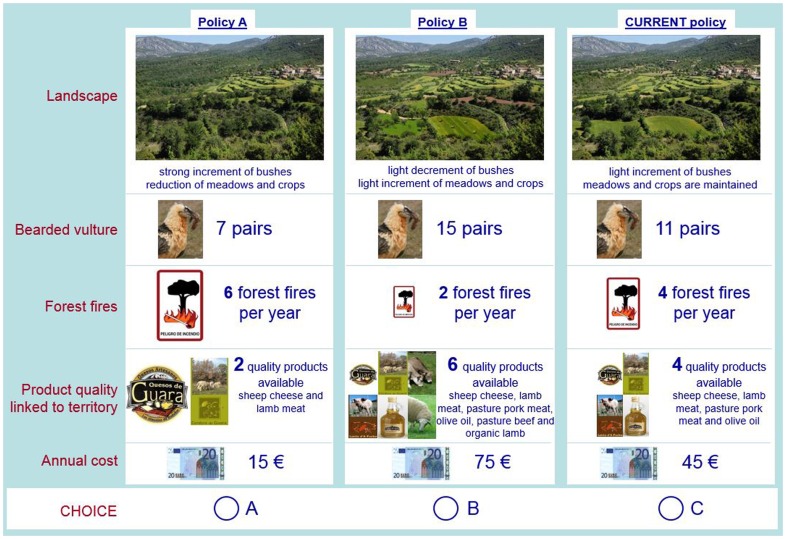
Choice set. For illustration, the attributes of policies A and B are represented with the levels corresponding to “liberalization” and “targeted support” policy scenarios (see File S1 for details). The actual choice sets presented to respondents use different combinations of attribute levels in policies A and B.

Respondents were asked to choose their most preferred policy scenario among three alternatives presented in the choice set. One of the alternatives was fixed (*status quo* situation) and corresponded to the current policy scenario. The other two alternatives were referred to as policy A and B and represented different combinations of attribute levels (Figure 1). The attribute levels were defined in biophysical terms according to contrasting policy scenarios called ‘liberalization’ and ‘targeted support’. The liberalization policy scenario assumes a reduction of support of both EU and national agri-environmental schemes. The targeted support policy scenario involves additional funding to agri-environmental schemes, which are specifically designed to deliver public goods (see File S1 for details).

Given the large number of combinations of attributes and levels (3 ∧ 4 * 5 ∧ 1 = 405) (Table 1), we used the software Ngene (Choice Metrics, Ltd.) to develop an efficient experiment design that included a fraction of these combinations. Thirty choice sets divided in six blocks were obtained, *i.e.*, each respondent made five choices. The design used prior parameter estimates obtained in a previous test survey (n = 70).

#### Survey and questionnaire

The survey was designed to collect the responses from the local population (residents in the SCGNP) and the general population in the region where the park is located (Aragón, Spain). For the general population, 402 persons over age 18 were interviewed through a professional online panel representative of the adult population in Aragón (N = 1103864) in June 2013. The panellists were recruited randomly by invitation, no voluntary registration was allowed ensuring the representativeness of the survey. For the local population (N = 934), 102 persons over age 18 were interviewed with a face-to-face interview in August and September 2013. Due to difficulties to ensure a probability sampling, judgement sampling based on age, gender and profession was carried out. Respondents were approached at their households or working sites.

The questionnaire had three parts. After explaining the purpose and geographical area of the study, and the structure of the questionnaire, 20 Likert-type questions were formulated on attitudes towards: the model of agriculture and food; the environment and economic development model; consumption and quality perception; and agri-environmental policies. These helped to investigate general societal perceptions (not presented in this paper) and familiarized respondents to the topic being evaluated in the choice experiment. The second part included the choice experiment. Before presenting the choice sets, a brief description of the mountain agroecosystems present in the area of study, the ES attributes, the agri-environmental policies in place, and the societal cost was presented to respondents. We explicitly made clear that the cost of each choice corresponded to the amount of money each member of the family above 18 would have to pay as an annual tax. The third part of the questionnaire collected socio-economic (age, gender, family size, education, income) and contextual (profession, agricultural background in the family, membership of consumer or nature associations, number of visits to the park and motives) information (see copy of questionnaire in File S2, in Spanish).

A face-to-face test interview to 70 non-randomly selected respondents was performed in March-April 2013. Some questions were reformulated afterwards to improve their understanding and results were analyzed to check for the coherence of the experiment design.

The Ethics Committee of the Centro de Investigación y Tecnología Agroalimentaria (Spain) approved the research protocol and questionnaire content. Anonymity of data was granted to participants in the survey, who expressed their consent to provide the information contained in the questionnaire.

#### Data analysis

Discrete choice methods are based on Random Utility Theory [29], which assumes individuals always choose the option that gives them the highest expected utility, and on the Theory of Value [30], where the utility or value obtained by individuals from a good or service is a function of its attributes or characteristics, and not only from of the good or service *per se*. This is relevant when valuing ES because most policy decisions do not involve a complete loss or gain in the provision of a particular ES but rather different levels of its provision. Choice models are able to estimate the level of utility or marginal value that an individual obtains from a particular good or service, defined by its attributes and levels. We used a mixed logit model, which allows for panel specification and unobserved preference heterogeneity among respondents, to analyse their choice. The utility function can be decomposed into deterministic (linear combination of observed attributes) and random components [31], where the latter capture both stochastic elements in the individuals' choices and individual utility elements not included in the deterministic part of the function. Furthermore, with the mixed logit specification we allow the effect of the explanatory attributes to vary among respondents. We used effects coding so that variables were not correlated with the grand mean of the utility function [27] to allow the calculation of WTP for all the levels [25].

## Results

### Socio-cultural valuation of ES

**Figure 2:**
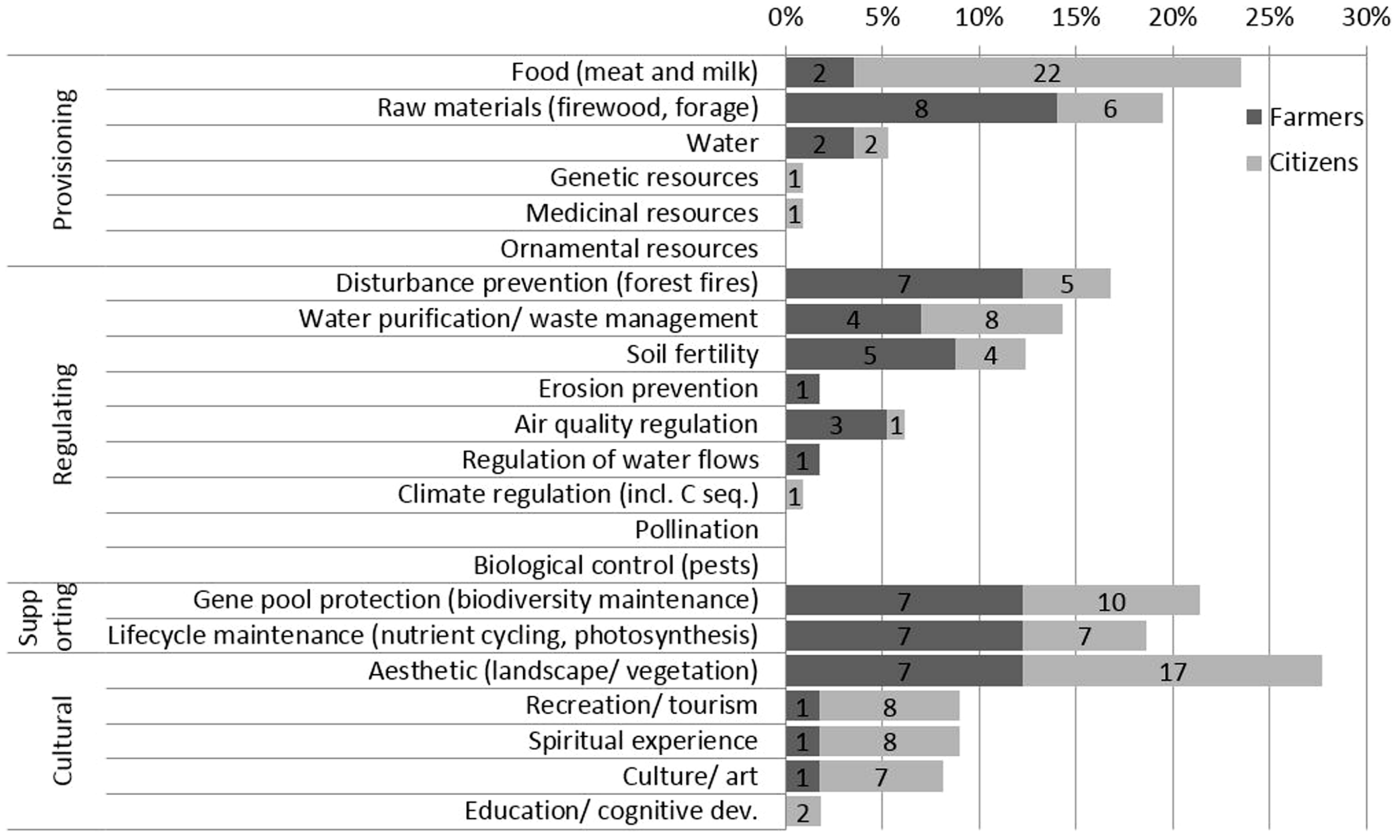
Percentage and number of times (within bars) that ecosystem services were mentioned during the FG with farmers and citizens. Note: modified from [23].

None of the participants in the FG discussions was familiar with the concept of ′ecosystem services′, but a number of them showed an intuitive understanding; some examples of definitions were “goods that nature provides to society”, “utility of diverse natural environments” or “economic benefits from nature”. Other participants interpreted the term as the responsibility of humans to preserve nature or could not interpret the term at all. Figure 2 shows the relative importance of individual ES according to the stakeholders, *i.e.*, farmers and citizens. Often, respondents did not use the same vocabulary as in the ES taxonomy, but the interpretation of transcripts allowed classifying different ideas into the ES typology, taking into consideration the contexts in which the discussions took place. Globally, the more frequent items mentioned were (in descending order): aesthetic (landscape/vegetation), provision of food (mainly discussed in terms of quality and safety of products), gene pool protection (biodiversity maintenance), lifecycle maintenance (nutrient cycling, photosynthesis), provision of raw materials (mainly forage and firewood), disturbance prevention (forest fires), water purification/waste management (always attached to industrial livestock systems as opposed to grazing ones), soil fertility/erosion prevention, and other cultural ES such as spiritual experience, recreation and culture.

Supporting ES were clearly identified by participants but were often expressed in a different manner to the formal terminology. For example, for lifecycle maintenance processes, participants used other terms that were familiar to them, such as “balance” or “equilibrium” between different components of nature. For gene pool protection, expressions such as diversity or number of “wild species” and “changes in local flora and fauna” were often used.

There were some differences between the perceptions of farmers and those of citizens. Farmers gave more importance (mentioned more times) to regulating ES, such as disturbance prevention (forest fires) and soil fertility/erosion prevention, the provision of raw materials, and supporting ES. Citizens gave more importance to all cultural ES, in particular opportunities for recreation, spiritual and cultural experiences and to the provision of food, mainly relating to quality and safety issues.

### Choice experiment

The most relevant ES per category identified above were considered in the choice experiment: cultural landscape, preservation of biodiversity, prevention of wild forest fires, and provision of local quality food products. Table 2 shows the results of the Mixed Logit model used to analyze the choice experiment data from the local (residents of the study area) and the general (region where the study area is located) populations. In the general sample, all attributes estimates are significant (most of them between 1 and 5% significance level). In the local sample, the estimate for the highest level of biodiversity was not significant. All parameter estimates showed the expected sign, i.e. a positive sign indicated a positive relationship between the independent and dependent variables, and a negative sign indicate a negative relationship. The highest level of forest fires showed high negative estimates, meaning that respondents in both populations strongly rejected (obtained welfare losses from) a higher number of forest fires, and *vice versa* (lowest level of forest fires showed similar estimates with a positive sign), all else being equal. Similarly, annual cost had a negative sign meaning that respondents preferred to pay lower taxes, all else being equal. The positive sign of the high level of quality products indicated that respondents preferred greater availability of products linked to the territory. However, the estimates for the low level of quality products were higher in absolute value, meaning that the welfare gains from avoiding the reduction in availability of quality products from the *status quo* situation was higher than the welfare gains of having more quality products available (especially in the general sample).

**Table 2:** Mixed Logit model results for the general and local samples.

	General sample				Local sample			
Parameter	Estimate^1^	Standard Error	t Value	P	Estimate^1^	Standard Error	t Value	P
Landsc. rich mosaic	0.3982	0.2171	1.83	0.0666	0.7428	0.2765	2.69	0.0072
Landsc. abandonment	−1.0471	0.3066	−3.41	0.0006	−0.7978	0.2873	−2.78	0.0055
Biodiversity 15	0.8877	0.3069	2.89	0.0038	0.2609	0.2144	1.22	0.2237
Biodiversity 7	−0.8434	0.2947	−2.86	0.0042	−0.5034	0.2406	−2.09	0.0364
Forest fires 6	−2.8342	0.9871	−2.87	0.0041	−1.4563	0.5573	−2.61	0.0090
Forest fires 2	2.5707	0.8265	3.11	0.0019	1.1894	0.3797	3.13	0.0017
Prod. quality 6	0.9789	0.4158	2.35	0.0186	0.7589	0.3405	2.23	0.0258
Prod. quality 2	−2.0904	0.7382	−2.83	0.0046	−1.1044	0.4721	−2.34	0.0193
Annual cost	−0.0399	0.0121	−3.30	0.0010	−0.0150	0.0082	−1.81	0.0697
*Model fit*								
No. respondents	402				102			
No. obs.	2010				510			
Log likelihood	−1892				−480.36			
McFadden LRI	0.1434				0.1427			

1 estimated regression coefficients that express the “marginal utility” of each attribute level.

In the general sample, the rich mosaic landscape level showed a positive but relatively low estimate in comparison to the level representing abandonment, which showed a large negative estimate. This indicated that the welfare gain from avoiding abandonment was much greater than the welfare gain of having more agricultural activity; this was not the case in the local sample, were both estimates were similar in absolute value. Likewise, a welfare gain from avoiding biodiversity loss was observed in the local sample, whereas the welfare gain of increasing it was not significant; this was not the case in the general sample, were both estimates had similar magnitudes in absolute value.

### Ranking and economic valuation of ES

The relative importance of each ES was established by calculating the Willingness to Pay (WTP) for each of the attributes included in the choice experiment. The partial WTP was calculated by dividing the absolute value of the estimates of the highest levels of the attributes (lowest for forest fires) by the absolute value of the estimate for the annual cost [25]. We calculated the Total Economic Value (TEV) by summing the partial WTP of attributes that corresponded to the different values of the TEV taxonomy (Table 1). As cost was included in the model as € person^−1^ year^−1^ so is also the WTP and TEV estimates. Table 3 shows that the most important ES (highest WTP) were the prevention of wild fires (53.2 and 40.3% of TEV) and the availability of quality products linked to the study area (20.2 and 25.7%) for the general and local samples, respectively. For the general sample, the next ES in importance were biodiversity (18.3%) and cultural landscape (8.2%), whereas for the local sample, it was the inverse, *i.e.*, 25.2% and 8.8% for cultural landscape and biodiversity, respectively. The TEV was greater for the local population than for the general sample (196.8€ person^−1^ year^−1^ vs. 121.2€, respectively).

**Table 3:** Willingness to Pay (WTP) (€ person^−1^ year^−1^) and composition of the Total Economic Value (TEV).

		General sample			Local sample		
ES	Value component of TEV	WTP	%	Rank	WTP	%	Rank
Landscape	Non-extractive direct use	10.0	8.2	4	49.5	25.2	3
Biodiversity	Non-use existence	22.2	18.3	3	17.4	8.8	4
Forest fires	Indirect use	64.4	53.2	1	79.3	40.3	1
Product Quality	Extractive direct use	24.5	20.2	2	50.6	25.7	2
TEV		121.2	100.0		196.8	100.0	

**Figure 3:**
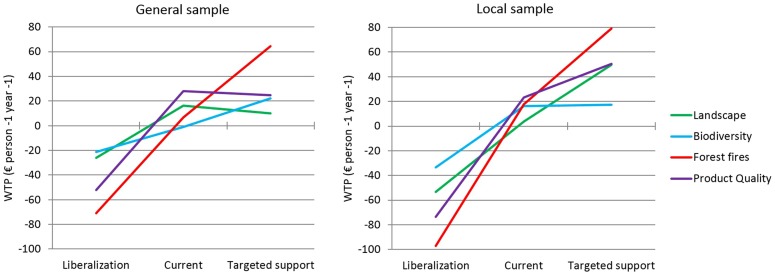
Willingness to Pay (WTP) (€ person^−1^ year^−1^) for ecosystem services in different policy scenarios.

Disaggregated WTP values for the different levels of the attributes are presented in Figure 3. In the general sample, the evolution of WTP from the liberalization to the targeted support scenarios was rather linear for forest fires and, with lower absolute values, for biodiversity. The pattern was different for availability of quality products and landscape, for which the WTP in the status quo scenario was slightly higher than in the targeted support scenario. In the local sample, forest fires and landscape showed a linear pattern, in contrast to availability of quality products and specially biodiversity.

## Discussion

The combination of socio-cultural and economic approaches constitutes a novel approach to quantify the multifunctionality of agriculture. In our study, the socio-cultural valuation was the basis of ES selection and choice design. People (stakeholders with different roles, from beneficiaries to payers of agri-environmental policies) defined the key functions (ES) under consideration. These, later defined in biophysical terms in the choice model, were integrated into the ES and TEV taxonomies, allowing for a holistic valuation of the ES (and their trade-offs) at different levels of multifunctionality (i.e. policy scenarios). To our knowledge, there is no similar approach in mountain areas of the Mediterranean basin and other European regions.

### Social perception of ES delivered by Mediterranean mountain agroecosystems

The fact that none of the FG participants were familiar with the “ecosystem service” concept questions its usefulness for communication with stakeholders, despite many studies underline the potential of the ES framework to influence policy design [32]. The non-provisioning ES to which FG participants gave higher importance were the maintenance of cultural landscape and natural vegetation (in relation to aesthetic value and recreational use value) and the prevention of wild forest fires. Together with biodiversity conservation (also highly mentioned during FG discussions), these three ES are considered inherently linked to certain types of agriculture predominant in European mountain areas (grazing livestock or mixed crop-livestock systems); the delivery of these ES, which constitute public goods, is very limited through alternative forms of land use [5]. These three ES have also received more attention in the scientific literature describing the biophysical relationships between grazing livestock systems and ES (see the review by [10]). Hence, there seems to be good correspondence between social perceptions and scientific focus for the multiple functions or ES provided by Mediterranean mountain agroecosystems.

The results indicated that farmers gave more importance to ES directly related to their own farming activity or to local circumstances or interests, whereas citizens showed more general concerns. Similar differences were described by Pereira et al. [33], Lamarque et al. [34], Martín-López et al. [35] and Oteros-Rozas et al. [36]. As stated by Daniel et al. [17], citizens clearly recognized the importance of cultural ES related to the aesthetic and recreational value of landscapes. In addition, cultural, spiritual and educational dimensions were also clearly identified (*e.g.*, traditional food and gastronomy, popular architecture, “old ways of living”, *etc.*). In general, cultural ES were often discussed in bundles [37] and often mixed with other types of ES, indicating a diverse understanding of multifunctionality that reflected the subjective backgrounds of the participants [38].

### Quantifying multifunctionality of Mediterranean mountain agroecosystems

Prevention of forest wildfires was the ES with highest importance (WTP). Wildfires and their associated impacts have dramatically increased in Spain and in other countries in the Euro-Mediterranean basin in the last few decades [4]. This result probably indicated a social perception of loss of value from indirect consumption of related provisioning ES (wood or pastures) and other ES (landscape, biodiversity, opportunities for recreation, *etc.*), but most importantly, explicitly showed how regulating ES can affect human well-being in terms of safety, security from disasters and adequate livelihoods [39].

The availability of specific quality products linked to the territory was second in importance. The demand for local and traditional food products has grown in many European countries in recent years [40], [41]. Although associated with extrinsic quality dimensions, such as heritage and culture, preservation of the environment, origin of the product, or specific processing, they can also be perceived as having superior sensory properties and higher safety standards [40], [42], [43]. Therefore, this ES can be considered as both a cultural and a provisioning (access to nutritious food and health) service.

Biodiversity and cultural landscape were ranked third and fourth in importance by the general population, with the inverse ranking for the local population. The comparatively low importance assigned to these ES could be because their consequences on human well-being are not immediate or not easily perceived. People tend to value more those ES that have direct effects and satisfy more tangible needs [44]. Additionally, the aesthetic perception of cultural landscapes is by definition subjective, and many respondents might not have been able to interpret the differences between the landscapes presented in the choice set or might not have valued as positive further human intervention in the *status quo* situation.

Large differences were found between the general and local populations. The TEV was significantly higher according to the local population (197€ person^−1^ year^−1^ *vs*. 120€ in the general population), with higher WTP for all components except for biodiversity. Two factors might explain this. On the one hand, an increasing level of ES provisioning (reduction of fires, higher possibilities for further development of food quality schemes or tourism industry) will directly affect the well-being of the local population [35]. On the other hand, respondents pursuing agricultural activities in their households are the direct beneficiaries of current agri-environmental programs. Local respondents showed lower WTP for biodiversity, possibly because they have a more functional interpretation of biodiversity (*e.g.*, conflicts between livestock and wild species) or they find the term elusive or even marginal [38]. The main difference, however, was observed for landscape. The WTP of local respondents was considerably higher; they clearly preferred a landscape with higher levels of human intervention and agricultural activity. These results suggest a positive link between respondents' attachment to the territory and the level of support for its landscape conservation [45] but also a higher appreciation of landscapes that provide higher levels of provisioning ES (agricultural products).

When disaggregating WTP at the different levels of ES supply (policy scenarios) trade-offs between ES became evident. Large welfare gains were observed for all ES in both populations when moving from the liberalization to the current scenario. However, when moving from the current to the targeted support scenarios, welfare gains due to further reduction of forest fires and higher biodiversity happened at the expense of the availability of quality products and a more human-intervened landscape in the general population. In the local population, welfare gains due to further reduction in the number of forest fires and more human-intervened landscape happened at the expense of availability of quality products and, more intensely, biodiversity. The asymmetry of welfare gains observed around the status quo scenario (Figure 3) is consistent with the large literature on loss aversion in behavioural economics, which shows that losses have greater impacts on preferences than gains [46].

### Implications for agri-environmental policy design

A paramount consideration of any ES valuation exercise is the purpose of the valuation [18]. In our case, the combination of qualitative (FG) and quantitative (economic valuation) research methods allowed the links between social and ecological systems to be visualized, aiming at integrating knowledge into policy design [17]. A good understanding of the social perception of values is required when designing agri-environmental policies to promote multifuncionality [47], taking into account the views of stakeholders with different roles and interests. Remarkably, although farmers/local residents and other citizens had divergent views and interests, they also shared a large number of concerns. The few ES showing higher levels of consensus (and the agricultural practices with greater potential to deliver them) could be targeted by agri-environmental policies in each particular agroecosystem; simple ad-hoc methodologies can be used to identify these ES.

In Europe, the CAP has failed in improving the delivery of public goods demanded by society. The choice of instruments, the design and implementation of policy measures and the distribution of budgetary resources figure among the main factors explaining this failure [5]. There is a need to regionalize and, if possible, individualize agri-environmental schemes at the farm level [10], and to establish concrete agricultural practices and environmental targets for the provision of non-market ES, considering existing scientific evidence to enable progress to be monitored. In the area of study, agricultural practices referring to adequate grazing pressure (number of animals grazing and duration of grazing season), the maintenance of mowing and diversification of forage crops (e.g. legumes) and the prevention of further abandonment of marginal areas should be prioritized. These practices have direct effects on shrub biomass and vegetation cover, and consequently on landscape quality, biodiversity and prevention of forest fires [22].

There is increasing evidence of large underestimation of the economic value of ES, the welfare loss linked to environmental degradation and the cost of inaction [11]. It is therefore necessary to value ES in monetary terms to allow farmers to be compensated in an equitable way for the public goods they deliver [48]. By individualizing support, monitoring and valuing objective indicators for ES and targeting particular agricultural practices, the so-called “green” subsidies of the CAP may truly become Payments for Ecosystem Services. There is a margin for this in the area of study, as the resulting WTP of the general population (121€ person^−1^ year^−1^) is threefold higher than the current level of support (45€ person^−1^ year^−1^ of cost of the current policy).

Finally, we should briefly mention some limitations of our study. First, some assumptions had to be made in terms of policies leading to certain levels of ES delivery. In addition, the indicators representing the ES could have been chosen differently, especially for landscape and biodiversity. A different definition of levels and indicators might have render different WTP and TEV values. However, File S1 offers a sound biophysical rationale for selecting the indicators and levels corresponding to the different scenarios in the choice model. We believe they are adequate for the purpose of the study. Second, the communication of ES and levels to the people is a major issue as we are valuing complex environmental phenomena. We cannot perfectly ascertain the understanding of ES or the rationale of people when making their choices, although mixed logit models try to address this issue. Yet, our results show consistent patterns. Third, a number of assumptions had to be made to calculate the TEV: i) we assumed the ES chosen represent well the different components of value in the TEV taxonomy, however, there is not a univocal relationship between specific ES and specific values and there is overlapping among ES and TEV components; ii) we did not consider option and bequest values (value of future use and value for future generations, respectively); iii) the TEV was calculated as the sum of the partial WTP of the ES in the targeted support scenario with respect to the status quo, however absolute values for WTP of ES in the liberalization scenario were larger and would have rendered higher TEV. Fourth, the results are space (and time) specific and only apply to a particular location. However, we think the results could, arguably, be scaled-up to wider Mediterranean mountain areas and rangelands in Europe.

## Conclusions

The combined use of deliberative and survey-based stated-preference methods enabled the links between social preferences, economic value and ecological systems to be visualized and quantified.

Cultural services have great importance for society, not only because of their aesthetic and recreational value but also for educational, cultural and spiritual reasons. Supporting services, essential for the delivery of all other ES, were also clearly recognized by farmers and other citizens. Although there were differences in perceptions between stakeholders according to their particular interests, they also shared common views for many ES.

In Mediterranean conditions, the prevention of forest fires is a key ES delivered by grazing agroecosystems. The production of specific quality products linked to the territory follows in importance as a key provisioning service. The maintenance of cultural landscape and biodiversity follow next, but are perceived differently by the local and general populations.

The willingness to pay for the provision of ES derived from Mediterranean mountain agroecosystems clearly exceeds the current level of public support in Europe. There is room to maneuver to enlarge the economic resources dedicated to agri-environmental schemes and to better target these schemes, allowing them to become Payments for Ecosystem Services.

## Supporting Information

File S1 **Detailed description of attributes and levels of the choice experiment.**
(DOCX)

File S2 **Questionnaire (block 1, in Spanish).**
(PDF)
