# Supplementary material for: Socio-Cultural and Economic Valuation of Ecosystem Services Provided by Mediterranean Mountain Agroecosystems
Source: PLoS One. 2014 Jul 18;9(7):e102479. doi: 10.1371/journal.pone.0102479 (PMC4103832; doi:10.1371/journal.pone.0102479)
Supplement: File S1 — Detailed description of attributes and levels of the choice experiment. (DOCX) [file pone.0102479.s001.docx]

Supporting Information S1. Detailed description of attributes and levels of the choice experiment

**1. Biophysical indicators for ES (attributes of the choice experiment)**

Agri-environmental indicators should convey relevant information about the ES in a particular location by being intuitive (communicating information about ecosystem services clearly and without ambiguity and being easily understood by the general public and policy makers), sensitive (able to detect changes in ecosystem status) and accepted (adhere to agreed scientific methods and available data sets) [[1](#_ENREF_1)]. A description of the biophysical indicators utilized to measure the ES identified in the socio-cultural valuation described in the paper follows. The assumptions used in classifying the ES into different components of TEV are also described.

***1.1 Cultural landscape***. The encroachment of vegetation is the main environmental problem associated with the abandonment of agricultural activities in mountain areas, with wide implications in terms of landscape [[2](#_ENREF_2)]. Some studies have shown a close link between grazing livestock and evolution of the vegetation in the Sierra y Cañones de Guara Natural Park (SCGNP). For example, [Riedel et al. [3]](#_ENREF_3), with data from 5 years of evolution of above-ground shrub biomass (kg DM/ha) in grazed and non-grazed areas, showed the differential effect of domestic grazing animals on the encroachment process (the rate of annual accretion of shrub biomass was 2563 kg/ha/year in non-grazed areas and 1173 kg/ha/year in grazed areas). According to [Ramírez et al. [4]](#_ENREF_4), the quality of rural landscapes can be assessed through a simplified model that takes into account four factors: vegetation, land use, form (elements differing from the background) and texture (reflection of light from surfaces). These 4 variables were used to modify the real image of a typical agricultural landscape in the area of study. The extent of forest and shrub cover, land use options (crops, mowing meadows and pastures), form (number and type of patches and differential elements, *i.e.*, hay bales) and texture (diverse land uses), can be modified to reflect hypothetical but realistic landscapes according to different land use scenarios.

Cultural landscape constitutes a cultural ES with a non-extractive direct use value, as it was identified in the socio-cultural valuation as an opportunity for recreation. However, it could also be related to other components of value, such as option value, bequest value and existence value.

***1.2 Biodiversity*** was assessed through an emblematic species of bird that relies intensely on domestic livestock, as it feeds on the carcasses of animals that remain in grazing areas [[5](#_ENREF_5)]. The Bearded Vulture (*Gypaetus barbatus*) is extinct in most mountain areas in Europe, except for the Pyrenees (147 pairs) including the SCGNP with 11 pairs, the Alps (21 pairs after a reintroduction programme), and Corsica and Crete (13 pairs) [[6](#_ENREF_6)]. It is considered “endangered of extinction” according to the Spanish List of Special Protection and Endangered Species (Regulation RD 139/2011) and is included in the Red Book of Spanish Birds and IUCN (International Union for Conservation of Nature) lists. The bearded vulture is a well-known threatened key-species. It can represent an actual or potential decline in biodiversity, and the recovery of a threatened species following management intervention is strongly indicative of successful conservation measures.

Biodiversity is a complex phenomenon with multiple roles in the delivery of many ES services, as a regulator of ecosystem processes, as an ES in itself and as a good [[7](#_ENREF_7)]. In our study, biodiversity is considered as an individual ES (supporting ES: gene pool protection/ biodiversity conservation), by definition basic for delivering many other ES. Due to the “intangibility” of the multiples roles of biodiversity for many people, we assume its represents well the non-use existence value in the TEV taxonomy (need of biodiversity preservation).

***1.3 Forest fires*.** The prevention of forest fires is considered a regulating service within the ES typology. The value of this ES could be calculated trough conventional methods (*i.e.*, cost of prevention and extinction); however, forest fires are very important in Mediterranean Areas and have enormous public and political concern. There is a strong link between the risk and scale of forest fires and the intensity of agro-silvo-pastoral activities (*i.e.*, grazing intensity related to encroachment) [[8](#_ENREF_8)]. As shown in the area of study and in neighbouring geographical areas (the Pyrenees), the variables that are mostly related to the occurrence of forest wildfires are the number of grazing days and the stocking rate [[3](#_ENREF_3),[9](#_ENREF_9)]. These last two studies were used to quantitatively define the risk of forest fires in terms of fire events for different scenarios, considering the average number of 4 forest fire events per year in the SCGNP.

The prevention of forest fires constitutes a regulating ES (regulation of environmental hazards) and therefore it has an indirect use value (indirect benefits of ecosystems functions).

***1.4 Quality products linked to the territory***. FG participants identified the availability of specific quality products linked to the territory as a very important ES provided by agriculture in the SCGNP. The demand for local and traditional food products has grown in many EU countries, notably in Euro-Mediterranean areas in recent years [[10](#_ENREF_10),[11](#_ENREF_11)]. These products tend to be associated with heritage and culture but also with product quality dimensions related to extrinsic attributes, such as preservation of the environment, origin or specific processing, and even intrinsic sensory properties [[10](#_ENREF_10),[12](#_ENREF_12),[13](#_ENREF_13)]. A variable number of quality products available to consumers in the park were considered in diverse scenarios. Currently, there are 4 local products with the corresponding private brands existing in the SCGNP: sheep cheese, lamb meat, pasture pork meat and olive oil.

We assumed that the availability of products with specific quality attributes linked to the SCGNP constitutes a provisioning ES (provision of food) with direct use value, transcending the consideration of these products as cultural ES (cultural heritage).

**2. Land use scenarios (levels of the choice experiment)**

Three land use scenarios (levels in the choice experiment) were defined: “current policy”, “liberalization of policy” and “targeted support” scenario, following the framework proposed by [Cooper et al. [14]](#_ENREF_14). The aim was to provide a qualitative framework within which to explore the provision of ES associated with agriculture in the SCGNP under a range of current or future policy settings. Scenarios can examine potential trends in agro-pastoral farming systems, agricultural land use and management practices, with implications for the provision of different types of ES [[14](#_ENREF_14)]. The social cost of alternatives or policy scenarios was defined according to the current support of agricultural policy for the provision of public goods, *i.e.*, the agri-environmental schemes of the Common Agricultural Policy or the European Union. Specifically, the budget of the Rural Development Program in the region was used to calculate the social cost (charge per person) of each scenario. The land use scenarios and consequences on provision of ES and cost are described in the box below.

| Scenario | Landscape | Biodiversity | Forest fires | Quality products | Cost |
| --- | --- | --- | --- | --- | --- |
| ***2.1 Current policy.*** The reference scenario assumes that European agricultural policy for rural development (Pillar 2) funds, in particular Axe 2 related to land management, remain stable. In this scenario, the number of farms and animals remains the same, and grazing management and other agricultural practices are also stable. Farms are very diverse in terms of intensification level, land use (and dependence on natural resources) and grazing management (*e.g.*, grazing season varied between 180 and 326 days) [[15](#_ENREF_15)]. However, current agri-environmental measures are implemented regardless of the real grazing management of the farm. As mentioned in the main text, current grazing pressure modulates but is not enough to stop the encroachment of shrub vegetation [[3](#_ENREF_3)]. | A real picture (2011) of Almunias de Rodellar village and its surroundings is presented. | According to [Lorente [6]](#_ENREF_6), there are 11 pairs of bearded vultures, and the population is considered stable in this scenario. | Under this scenario the risk of forest fires and therefore the number of events remains moderate, at in 4 forest fires per year (average annual number of fire events in the period 1974-1997) | The number of quality products available to consumers in the park is 4, corresponding to private brands for: sheep cheese, lamb meat, pasture pork meat and olive oil. | The yearly budget of the RDP of Aragón (2007-2013) in the Axe 2 (Improvement of the Environment) was divided by the number of inhabitants above 18, resulting in a cost of 45.5€ per person per year. |
| ***2.2 Liberalization of policy.*** This hypothetical scenario depicts the ‘liberalization’ of agricultural policy, and therefore assumes a reduction of support in the form of both EU and national agri-environmental measures. Therefore, there is a process of reduction of agriculture, or even abandonment of agriculture in some areas. It is very difficult to forecast the evolution of the agricultural sector in the area of study with a scenario of drastic reduction of subsidies. According to data from [Pardos et al. [16]](#_ENREF_16), 66% of sheep farms in a representative sample in the region obtain a negative Gross Margin before premiums. We have assumed that the decrease of Common Agricultural Policy premiums would cause a decrease in animals and farms, especially those with already uncertain continuity prospects (38% of farms in the PNSCG) that have a grazing management regime considered more beneficial for conservation of the park [[15](#_ENREF_15)]. Some of the remaining farms could develop more “extensive” grazing management to reduce costs; however, grazing pressure diminishes considerably and large pastoral areas are abandoned in the near future under this scenario. | An alternative picture is presented showing more marginal areas abandoned and shrub and forest vegetation increases. Cultivation is reduced, and grazing-only meadows predominate. The landscape structure is more homogeneous (reduction in the number and types of patches). | [Margalida et al. [5]](#_ENREF_5) estimated that the decline in the population of bearded vultures in a similar area caused by reduction of biomass from domestic animals would mean that the species decrease drastically (from 16 to 6 pairs) and would not recover. | [Tous de Sousa [17]](#_ENREF_17) indicated that the surface affected by forest fires in a no-intervention scenario increases 313.5%. [Vicente-Serrano et al. [9]](#_ENREF_9) indicated an increase of fire risk of 27.1% between 1957 and 2000 because of agricultural abandonment and lack of livestock grazing. We considered that the number of fire events increases by 50%, *i.e.*, 6 fires per year. | The number of quality products available to consumers decreases to 2 as a consequence of the reduction of support. | The specific policies for maintaining/ improving ecosystem services in this alternative have a much lower budget, and therefore the societal cost per person decreases to 15€ per year.  It should be noted that cost is introduced as a continuous variable in the choice experiment to be able to estimate WTP. |
| ***2.3 Targeted support***. This scenario involves the greening of the Common Agricultural Policy through the more targeted support of agri-environmental schemes, which would have a positive effect on biodiversity and on the ES derived from agriculture; this impact can be attributed to an increase in extensively managed grasslands. There are supplementary payments in areas of disadvantage or natural handicap, and the Pillar 2 budget increases. Most measures are specifically designed and targeted to the delivery of public goods, and concrete targets are established so that agri-environmental measures become Payments for Ecosystems Services, in which farmers are compensated for the ES they deliver. As a consequence, even if the total number of farms and animals does not vary under this scenario, land use is modified (recovery of some previously abandoned areas, recovery of agricultural and forage crops, mowing meadows) and grazing management is targeted towards the provision of ecosystem services, *i.e.*, more animals are grazing for longer periods of time, covering key areas where grazing was abandoned in the past. | An alternative picture shows a situation where some abandoned areas are cultivated again and land use is more diversified (different agricultural crops, *e.g.*, cereals, trees; mowing meadows and forage crops, *e.g.*, hay bales). The landscape structure is diversified (increase in the number and types of patches), emulating a rich mosaic. | According to [Lorente [6]](#_ENREF_6), the population of bearded vultures might not increase considerably in the PNSCG even if more feed was available, as this species is close to the carrying capacity. However, we have considered that the population can still grow to 15 pairs to reflect the fact that some pairs can colonize suitable neighbouring areas [[18](#_ENREF_18)]. | [Tous de Sousa [17]](#_ENREF_17) indicated that the surface affected by forest fires in an optimal intervention scenario decreases by 71.5%. We considered that the number of fire events decreases by 50%, *i.e.*, 2 fires per year. | The number of quality products available to consumers increases to 6, including pasture beef and organic lamb. | The specific policies for maintaining/ improving ecosystem services in this alternative have a larger budget, and so the societal cost per person increases to 75€ per year.  It should be noted that cost is introduced as a continuous variable in the choice experiment to be able to estimate WTP. |

**References of Supporting Information S1**

1. Layke K (2009) Measuring Nature's Benefits: A Preliminary Roadmap for Improving Ecosystem Service Indicators. Washington: World Resources Institute (WRI). 36 p.

2. Lasanta-Martínez T, Vicente-Serrano SM, Cuadrat-Prats JM (2005) Mountain Mediterranean landscape evolution caused by the abandonment of traditional primary activities: a study of the Spanish Central Pyrenees. Appl Geogr 25: 47-65.

3. Riedel JL, Bernues A, Casasus I (2013) Livestock Grazing Impacts on Herbage and Shrub Dynamics in a Mediterranean Natural Park. Rangeland Ecol Manag 66: 224-233.

4. Ramírez Á, Ayuga-Téllez E, Gallego E, Fuentes JM, García AI (2011) A simplified model to assess landscape quality from rural roads in Spain. Agric, Ecosyst Environ 142: 205-212.

5. Margalida A, Colomer MA, Sanuy D (2011) Can wild ungulate carcasses provide enough biomass to maintain avian scavenger populations? An empirical assessment using a bio-inspired computational model. PLoS ONE 6: e20248.

6. Lorente L (2010) Sierra de Guara: quebrantahuesos con sello humano. Querqus. pp. 16-23.

7. Mace GM, Norris K, Fitter AH (2012) Biodiversity and ecosystem services: A multilayered relationship. Trends Ecol Evol 27: 19-25.

8. Lasanta-Martínez T, González-Hidalgo JC, Vicente-Serrano SM, Sferi E (2006) Using landscape ecology to evaluate an alternative management scenario in abandoned Mediterranean mountain areas. Landscape Urban Plan 78: 101-114.

9. Vicente-Serrano SM, Lasanta-Martinez T, Cuadrat-Prats JM (2000) Influencia de la ganadería en la evolución del riesgo de incendio en función de la vegetación en un área de montaña: el ejemplo del valle de Borau (Pirineo aragonés). Geographicalia 38: 33-57.

10. Lenglet F (2014) Influence of terroir products meaning on consumer’s expectations and likings. Food Qual Preference 32, Part C: 264-270.

11. Guerrero L, Claret A, Verbeke W, Enderli G, Zakowska-Biemans S, et al. (2010) Perception of traditional food products in six European regions using free word association. Food Qual Preference 21: 225-233.

12. Guerrero L, Guàrdia MD, Xicola J, Verbeke W, Vanhonacker F, et al. (2009) Consumer-driven definition of traditional food products and innovation in traditional foods. A qualitative cross-cultural study. Appetite 52: 345-354.

13. Bernués A, Riedel JL, Casasús I, Olaizola A (2006) The conservation of natural resources as an extrinsic quality attribute of lamb in Mediterranean Areas. In: Ramalho Ribeiro JMC, Horta AEM, Mosconi C, Rosati A, editors. Animal products from the Mediterranean area. Wageningen: Wageningen Academic Publishers. pp. 73-82.

14. Cooper T, Hart K, Baldock D (2009) Provision of Public Goods through Agriculture in the European Union. London: Institute for European Environmental Policy. 396p.

15. Riedel JL, Casasús I, Bernués A (2007) Sheep farming intensification and utilization of natural resources in a Mediterranean pastoral agro-ecosystem. Livest Sci 111: 153-163.

16. Pardos L, Maza MT, Fantova E, Sepúlveda W (2008) The diversity of sheep production systems in Aragón (Spain): characterisation and typification of meat sheep farms. Span J Agric Res 6: 497-507.

17. Tous de Sousa C (2010) Alternativas de gestión del riesgo de incendio forestal en el marco del desarrollo rural: Centro Internacional de Altos Estudios Agronómicos Mediterráneos - Instituto Agronómico Mediterráneo de Zaragoza (CIHEAM-IAMZ). 72 p.

18. Heredia R (2005) Status y distribución del quebrantahuesos en España y diagnóstico de la situación de la población en la UE In: Margalida A, Heredia R, editors. Biología de conservación del Quebrantahuesos (Gypaetus barbatus) en España. Madrid: Organismo Autónomo de Parque Nacionales. pp. 21-37.
