# Supplementary material for: Socio-Cultural and Economic Valuation of Ecosystem Services Provided by Mediterranean Mountain Agroecosystems
Source: PLoS One. 2014 Jul 18;9(7):e102479. doi: 10.1371/journal.pone.0102479 (PMC4103832; doi:10.1371/journal.pone.0102479)
Supplement: File S2 — Questionnaire (block 1, in Spanish). (PDF) [file pone.0102479.s002.pdf]

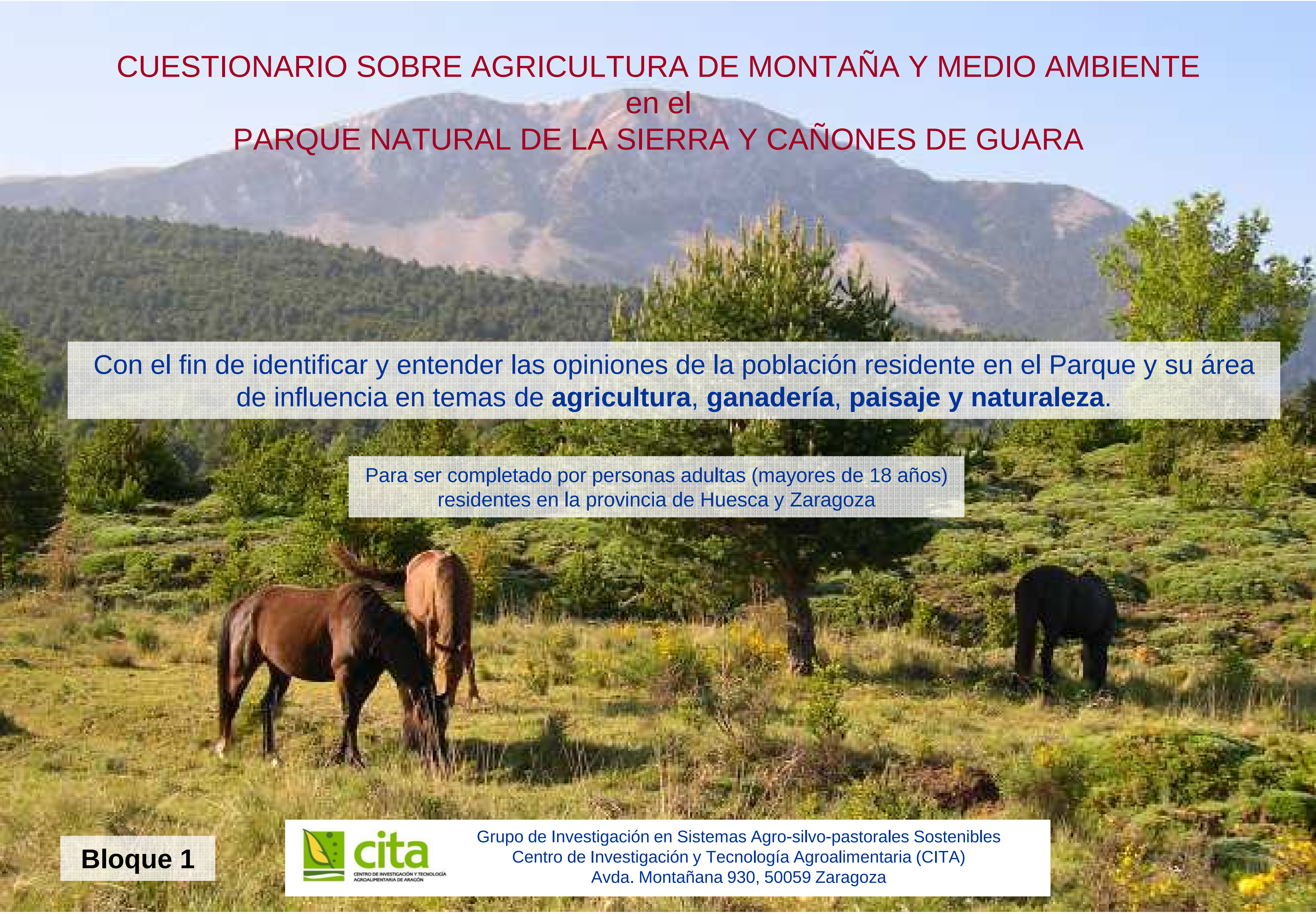

CUESTIONARIO SOBRE AGRICULTURA DE MONTAÑA Y MEDIO AMBIENTE en el PARQUE NATURAL DE LA SIERRA Y CAÑONES DE GUARA

Con el fin de identificar y entender las opiniones de la población residente en el Parque y su área de influencia en temas de **agricultura**, **ganadería**, **paisaje y naturaleza**.

Para ser completado por personas adultas (mayores de 18 años) residentes en la provincia de Huesca y Zaragoza

Bloque 1

cita
CENTRO DE INVESTIGACIÓN Y TECNOLOGÍA AGROALIMENTARIA DE ARAGÓN

Grupo de Investigación en Sistemas Agro-silvo-pastorales Sostenibles
Centro de Investigación y Tecnología Agroalimentaria (CITA)
Avda. Montañana 930, 50059 Zaragoza

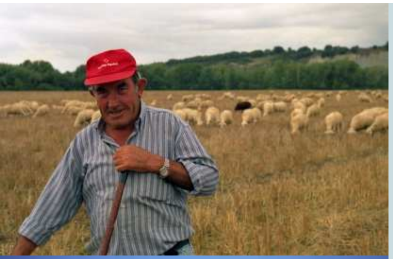
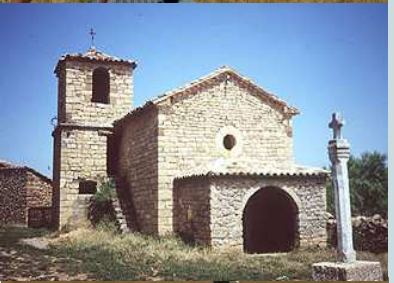
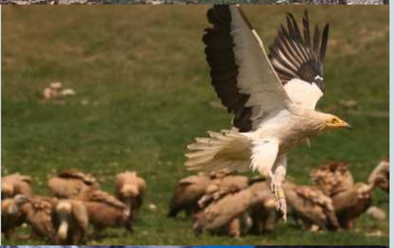
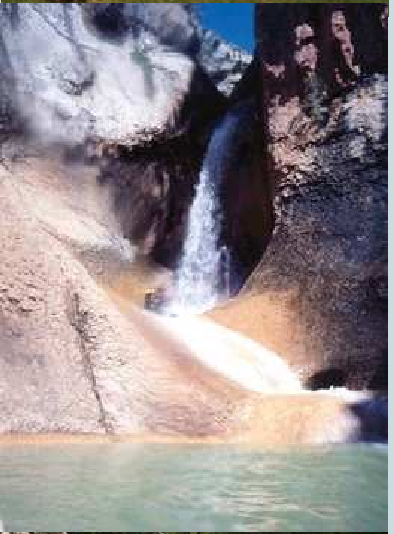
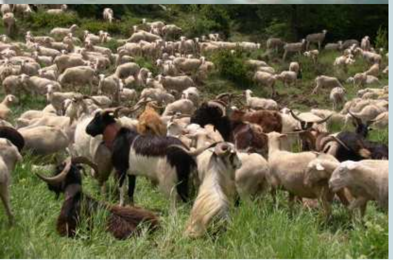

La **agricultura y ganadería** en zonas de montaña, como el Parque Natural de la Sierra y Cañones de Guara en Huesca, cumplen un papel muy importante en la producción de **alimentos de calidad**, contribuyendo a mantener la actividad económica en este **medio rural**.

La actividad agraria resulta también fundamental en el mantenimiento de los **paisajes** tradicionales del Parque, la conservación de gran **diversidad de plantas y animales**, o la prevención de **incendios forestales**.

Sin embargo, este tipo de **agricultura tradicional ha disminuido** o incluso desaparecido completamente en algunas zonas del Parque, por lo que estos **valores naturales** se están viendo **amenazados**.

Este cuestionario está dividido en **3 partes** y trata de **conocer la opinión** de los habitantes del Parque y zonas de influencia (Huesca y Zaragoza) sobre estos temas.

El **objetivo** de la encuesta es orientar mejor las **políticas agrarias y medioambientales** del Gobierno de Aragón y la Unión Europea en este territorio.

Su **participación** nos resulta **muy valiosa**. Le pedimos que dedique unos minutos de su tiempo para completar el cuestionario.

LA INFORMACIÓN ES ABSOLUTAMENTE CONFIDENCIAL Y NO SE UTILIZARÁ FUERA DE LOS OBJETIVOS DEL TRABAJO, NI SE HARÁN PÚBLICOS LOS DATOS INDIVIDUALES DE LOS PARTICIPANTES.

Este trabajo forma parte de 2 proyectos de investigación desarrollados por el CITA en colaboración con otros institutos de investigación españoles y europeos, y está financiado por:

- Instituto Nacional de Investigación y Tecnología Agraria y Alimentaria (INIA)
- Comisión Europea (7º Programa Marco de Investigación)

INIA
Instituto Nacional de Investigación y Tecnología Agraria y Alimentaria

European Commission

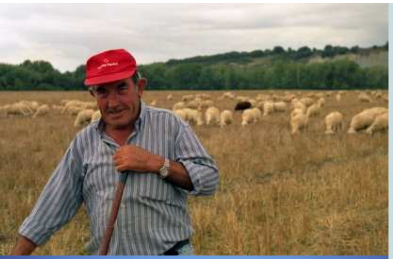
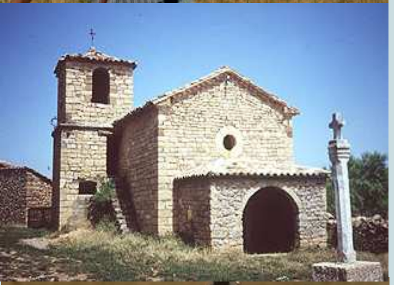
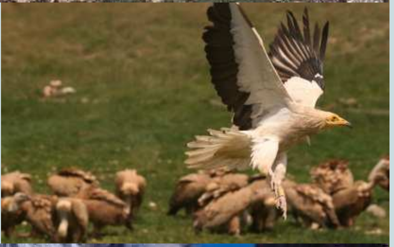
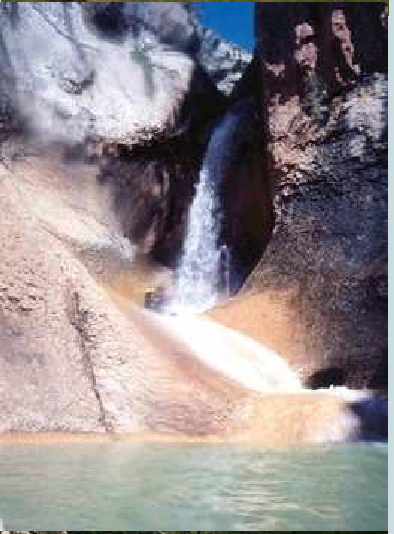
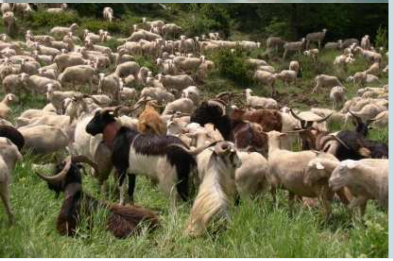

Parte 1. Grado de acuerdo o desacuerdo

Por favor, marque la casilla que mejor refleje su postura en cada una de las siguientes afirmaciones:

1	2	3	4	5
Totalmente en desacuerdo	En desacuerdo	Neutral	De acuerdo	Totalmente de acuerdo

	1	2	3	4	5
1. Hay que cambiar el modelo de desarrollo económico e integrar la conservación del medio ambiente	○	○	○	○	○
2. Los supermercados y grandes superficies ofrecen mayores garantías de calidad que las tiendas tradicionales	○	○	○	○	○
3. Si pudiera elegir, preferiría vivir en el campo que en la ciudad	○	○	○	○	○
4. Los recursos naturales están a nuestro servicio y debemos sacar el máximo provecho de ellos	○	○	○	○	○
5. Las nuevas tecnologías de transformación y envasado de alimentos aumentan la calidad de los productos	○	○	○	○	○
6. Deben acentuarse los esfuerzos para detener el abandono del medio rural	○	○	○	○	○
7. Los productos ecológicos, de temporada, y con origen local son una alternativa de consumo más justa y sostenible	○	○	○	○	○
8. Las subvenciones agrarias deben concederse a todos los agricultores y ganaderos según su nivel de producción	○	○	○	○	○
9. Es necesario mejorar el control en la aplicación de las políticas agrarias y medioambientales	○	○	○	○	○
10. La agricultura intensiva (industrializada) es la mejor manera de solucionar el hambre en el mundo	○	○	○	○	○

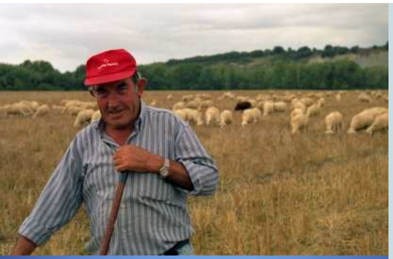
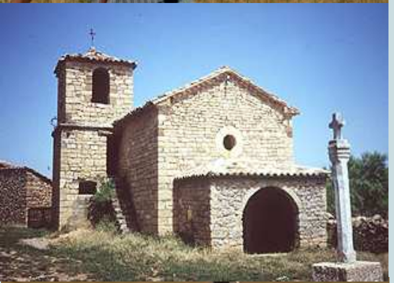
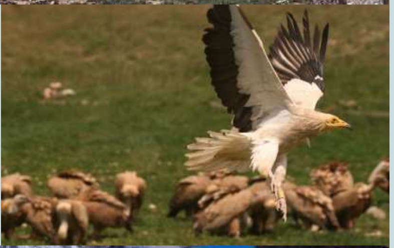
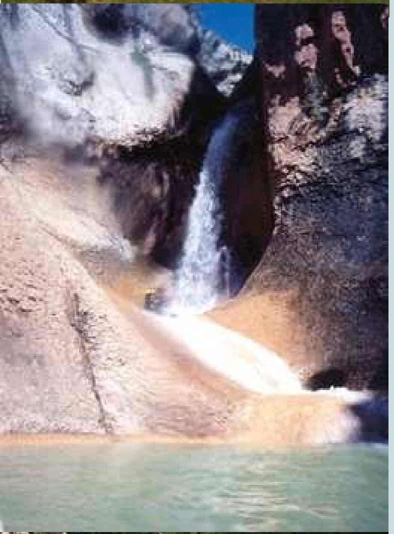
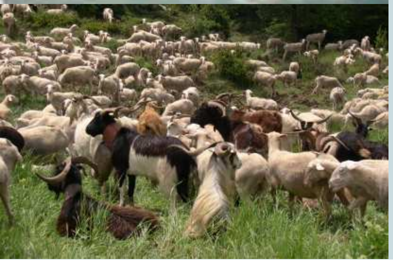

Parte 1. Grado de acuerdo o desacuerdo

Por favor, marque la casilla que mejor refleje su postura en cada una de las siguientes afirmaciones:

1	2	3	4	5
Totalmente en desacuerdo	En desacuerdo	Neutral	De acuerdo	Totalmente de acuerdo

Afirmación	1	2	3	4	5
11. Cuando salgo al campo prefiero ver paisajes vírgenes, no intervenidos por el hombre (ej. alta montaña)	○	○	○	○	○
12. El Gobierno debe reducir los recursos económicos dedicados a políticas ambientales y dedicarlos a otras políticas	○	○	○	○	○
13. La ganadería siempre tiene un impacto ambiental negativo	○	○	○	○	○
14. El cambio climático es uno de los problemas más importantes a los que se enfrenta la sociedad actual	○	○	○	○	○
15. Los agricultores y ganaderos que se localizan en zonas menos desarrolladas y con altos valores naturales y turísticos deberían recibir mayores subvenciones	○	○	○	○	○
16. Deben mantenerse las políticas agrarias y subvenciones actuales puesto que la agricultura es un sector estratégico	○	○	○	○	○
17. Los supermercados y grandes superficies ofrecen mayores garantías de seguridad alimentaria que las tiendas tradicionales	○	○	○	○	○
18. El crecimiento económico es más importante que la conservación de la naturaleza	○	○	○	○	○
19. Deberíamos cambiar nuestra forma de vida: debemos consumir menos	○	○	○	○	○
20. Procuro informarme de la manera en que se producen los alimentos y su origen	○	○	○	○	○

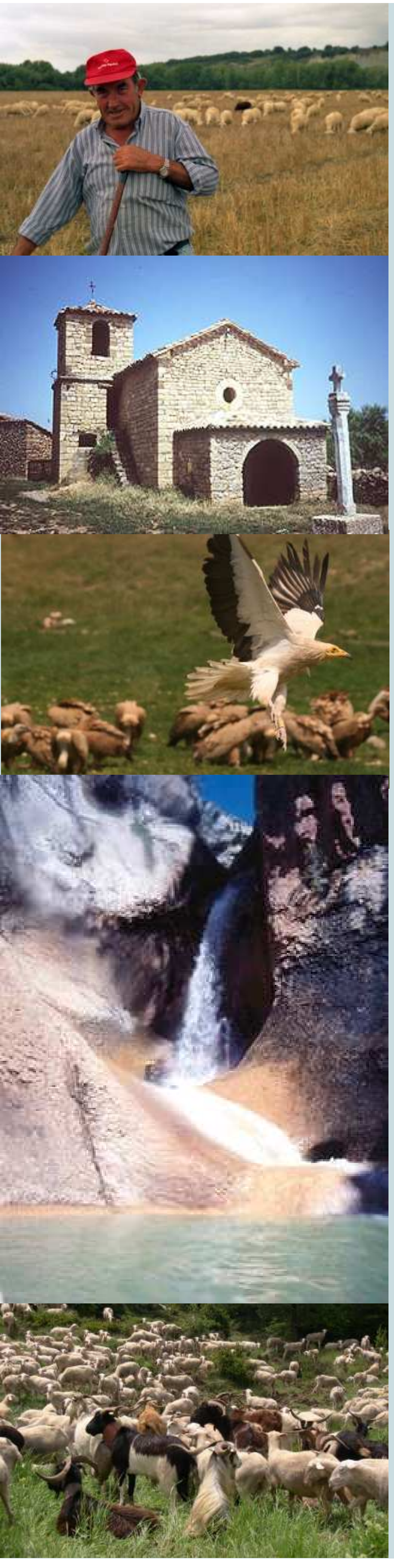

Parte 2. Cinco fichas (por favor, lea este breve texto con atención antes de contestar)

A continuación le vamos a presentar **5 fichas**.

Cada ficha presenta 3 **políticas agro-ambientales** definidas por una combinación de características en el Parque Natural de Guara: **paisaje tradicional, conservación de especies amenazadas** como el Quebrantahuesos**, prevención de incendios** y disponibilidad de **alimentos de calidad** ligados al territorio.

En cada ficha, la tercera columna corresponde a la **política que se da actualmente** que tiene un coste de 45€ por persona adulta y año. El Gobierno está valorando otras políticas agro-ambientales alternativas en este tipo de espacios. Opciones más caras y más baratas serán consideradas.

El **coste** corresponde a la cantidad que **cada miembro de su hogar** mayor de edad deberá pagar anualmente en su declaración de la renta para financiar la política elegida.

En cada ficha usted debe elegir la opción (A, B ó C) que prefiere

Bloque 1
Ficha 2

	Política A	Política B	Política ACTUAL
Paisaje<br>clicar sobre la imagen para ampliar	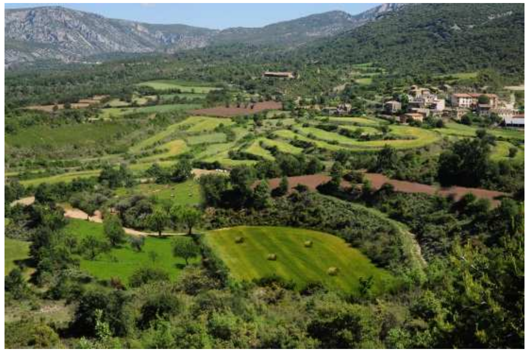<br>ligera reducción de matorral,<br>ligero incremento de praderas y cultivos	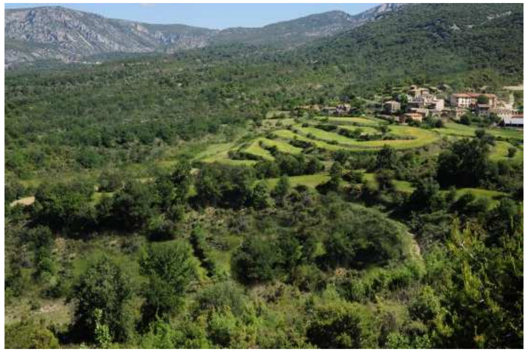<br>fuerte incremento de matorral,<br>reducción de praderas y cultivos	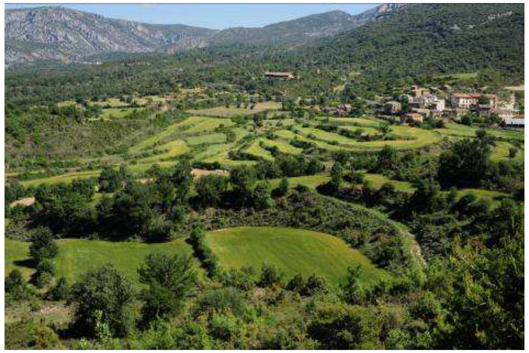<br>ligero incremento de matorral,<br>mantenimiento de praderas y cultivos
Quebrantahuesos	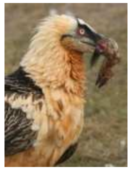 15 parejas	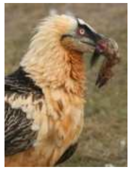 11 parejas	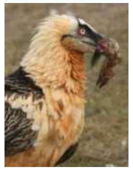 11 parejas
Incendios forestales	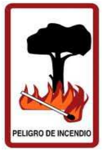 4 incendios al año	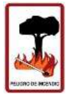 2 incendios al año	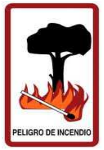 4 incendios al año
Productos de calidad ligados al territorio	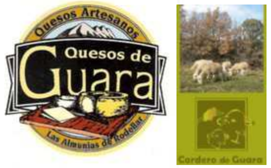 **2** productos de calidad disponibles<br>queso de oveja y carne de cordero	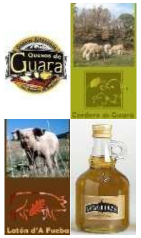 **4** productos de calidad disponibles<br>queso de oveja, carne de cordero, carne de cerdo extensivo y aceite de oliva	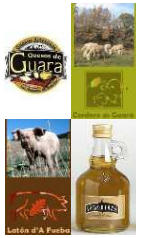 **4** productos de calidad disponibles<br>queso de oveja, carne de cordero, carne de cerdo extensivo y aceite de oliva
Coste anual	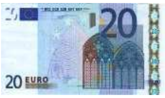 45 euros	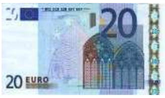 60 euros	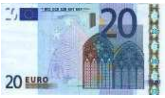 45 euros
OPCION ELEGIDA	○ A	○ B	○ C

Bloque 1
Ficha 6

	Política A	Política B	Política ACTUAL
Paisaje<br>clicar sobre la imagen para ampliar	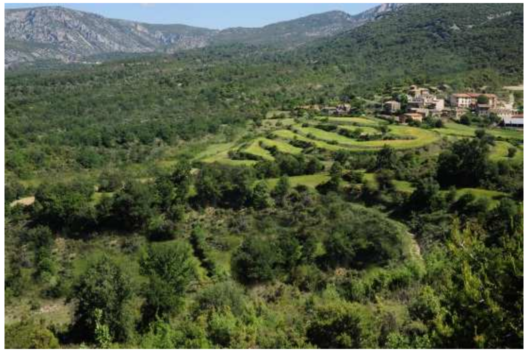<br>fuerte incremento de matorral,<br>reducción de praderas y cultivos	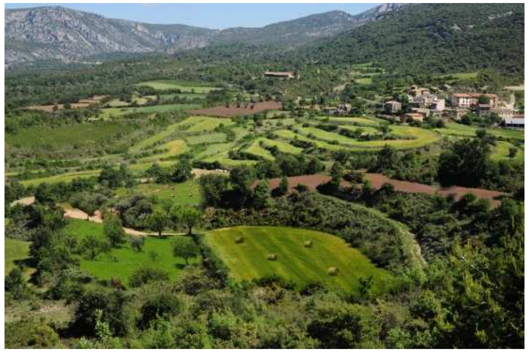<br>ligera reducción de matorral,<br>ligero incremento de praderas y cultivos	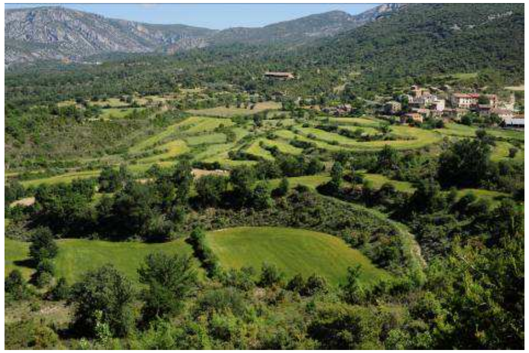<br>ligero incremento de matorral,<br>mantenimiento de praderas y cultivos
Quebrantahuesos	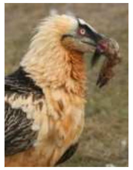 7 parejas	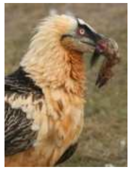 15 parejas	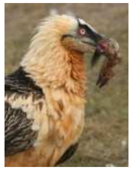 11 parejas
Incendios forestales	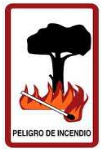 4 incendios al año	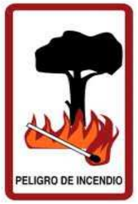 6 incendios al año	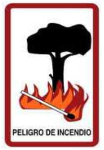 4 incendios al año
Productos de calidad ligados al territorio	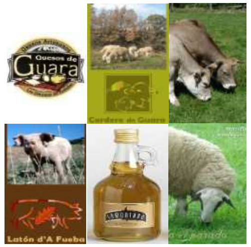 **6** productos de calidad disponibles<br>queso de oveja, carne de cordero, carne de cerdo extensivo, aceite de oliva, carne de novillo extensivo y masito (cordero ecológico)	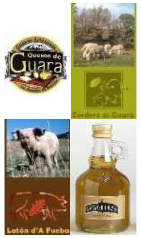 **4** productos de calidad disponibles<br>queso de oveja, carne de cordero, carne de cerdo extensivo y aceite de oliva	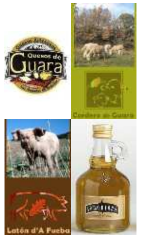 **4** productos de calidad disponibles<br>queso de oveja, carne de cordero, carne de cerdo extensivo y aceite de oliva
Coste anual	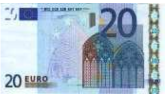 30 euros	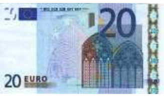 45 euros	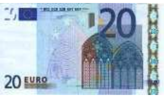 45 euros

OPCION ELEGIDA ○ A ○ B ○ C

Bloque 1
Ficha 8

	**Política A**	**Política B**	**Política ACTUAL**
Paisaje<br>clicar sobre la imagen para ampliar	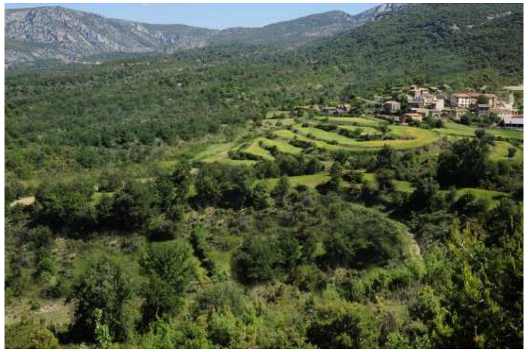<br>fuerte incremento de matorral,<br>reducción de praderas y cultivos	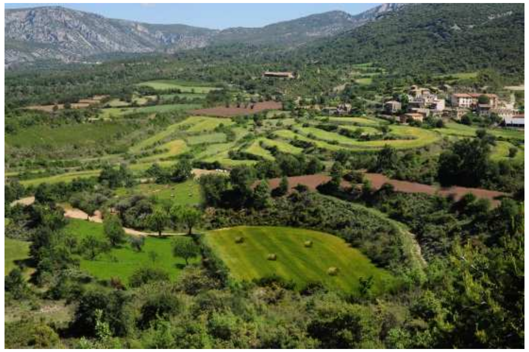<br>ligera reducción de matorral,<br>ligero incremento de praderas y cultivos	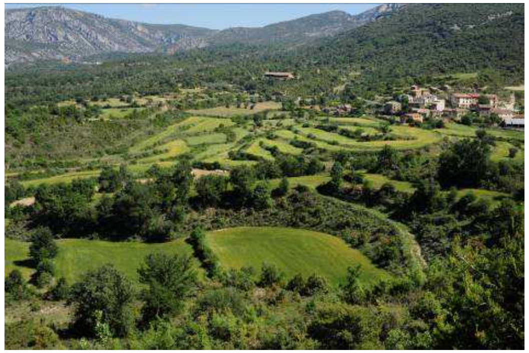<br>ligero incremento de matorral,<br>mantenimiento de praderas y cultivos
Quebrantahuesos	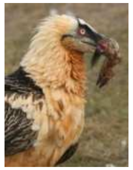 7 parejas	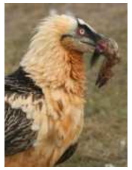 7 parejas	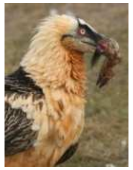 11 parejas
Incendios forestales	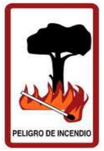 4 incendios al año	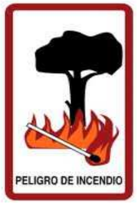 6 incendios al año	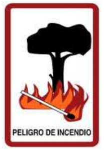 4 incendios al año
Productos de calidad ligados al territorio	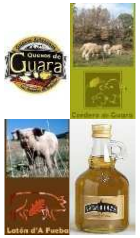 **4** productos de calidad disponibles<br>queso de oveja, carne de cordero, carne de cerdo extensivo y aceite de oliva	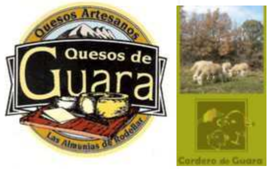 **2** productos de calidad disponibles<br>queso de oveja y carne de cordero	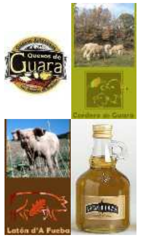 **4** productos de calidad disponibles<br>queso de oveja, carne de cordero, carne de cerdo extensivo y aceite de oliva
Coste anual	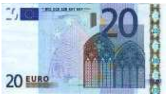 60 euros	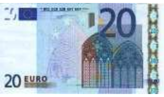 45 euros	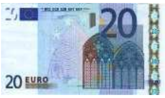 45 euros

OPCION ELEGIDA ◯ A ◯ B ◯ C

Bloque 1
Ficha 21

	Política A	Política B	Política ACTUAL
Paisaje<br>clicar sobre la imagen para ampliar	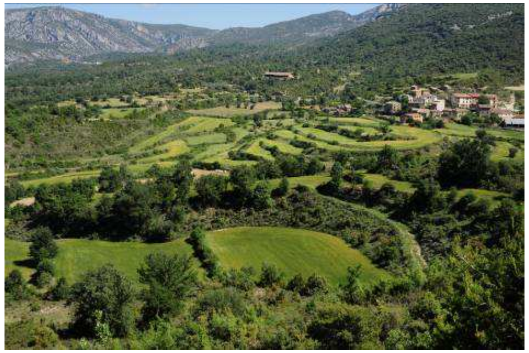<br>ligero incremento de matorral, mantenimiento de praderas y cultivos	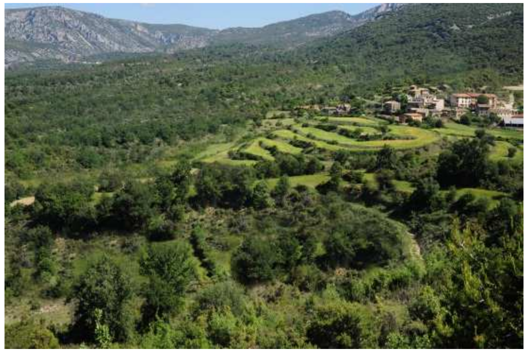<br>fuerte incremento de matorral, reducción de praderas y cultivos	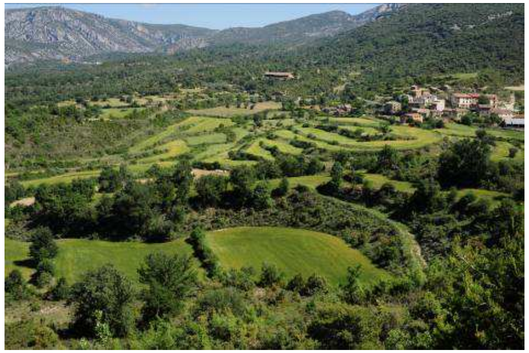<br>ligero incremento de matorral, mantenimiento de praderas y cultivos
Quebrantahuesos	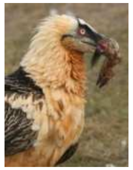 15 parejas	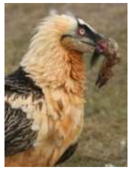 11 parejas	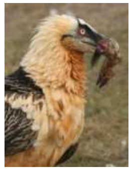 11 parejas
Incendios forestales	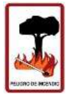 2 incendios al año	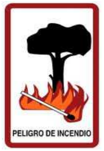 4 incendios al año	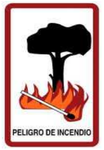 4 incendios al año
Productos de calidad ligados al territorio	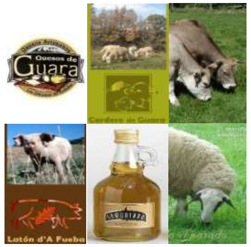 **6** productos de calidad disponibles<br>queso de oveja, carne de cordero, carne de cerdo extensivo, aceite de oliva, carne de novillo extensivo y masito (cordero ecológico)	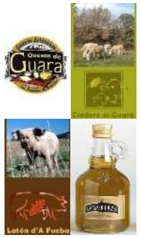 **4** productos de calidad disponibles<br>queso de oveja, carne de cordero, carne de cerdo extensivo y aceite de oliva	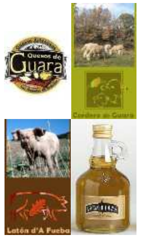 **4** productos de calidad disponibles<br>queso de oveja, carne de cordero, carne de cerdo extensivo y aceite de oliva
Coste anual	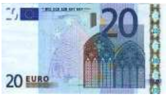 75 euros	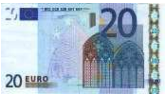 15 euros	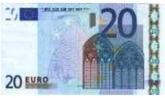 45 euros

OPCION ELEGIDA ○ A ○ B ○ C

Bloque 1
Ficha 23

	**Política A**	**Política B**	**Política ACTUAL**
Paisaje<br>clicar sobre la imagen para ampliar	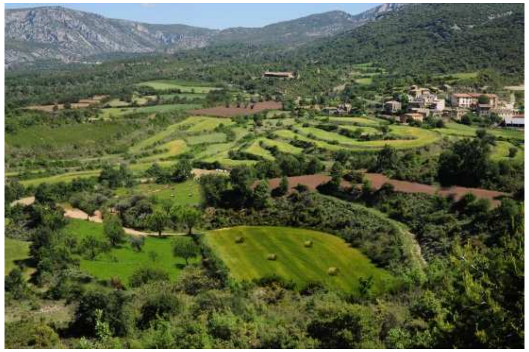<br>ligera reducción de matorral,<br>ligero incremento de praderas y cultivos	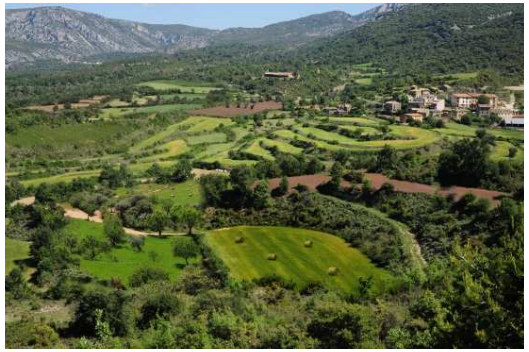<br>ligera reducción de matorral,<br>ligero incremento de praderas y cultivos	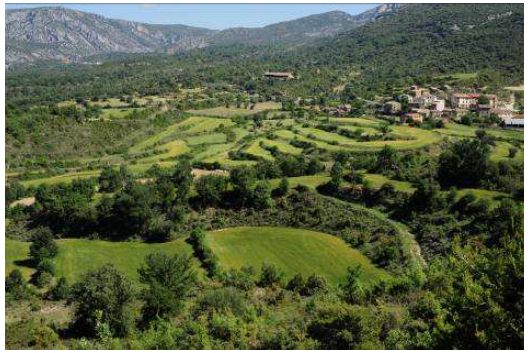<br>ligero incremento de matorral,<br>mantenimiento de praderas y cultivos
Quebrantahuesos	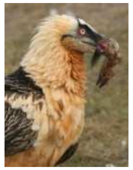 7 parejas	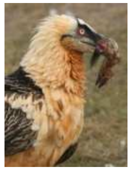 7 parejas	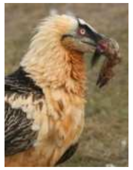 11 parejas
Incendios forestales	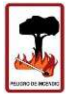 2 incendios al año	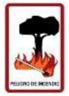 2 incendios al año	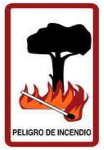 4 incendios al año
Productos de calidad ligados al territorio	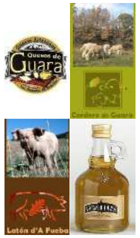 **4** productos de calidad disponibles<br>queso de oveja, carne de cordero, carne de cerdo extensivo y aceite de oliva	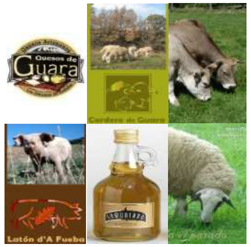 **6** productos de calidad disponibles<br>queso de oveja, carne de cordero, carne de cerdo extensivo, aceite de oliva, carne de novillo extensivo y masito (cordero ecológico)	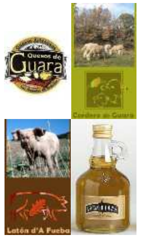 **4** productos de calidad disponibles<br>queso de oveja, carne de cordero, carne de cerdo extensivo y aceite de oliva
Coste anual	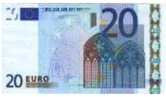 45 euros	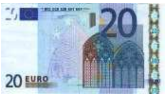 75 euros	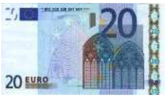 45 euros
OPCION ELEGIDA	◯ A	◯ B	◯ C

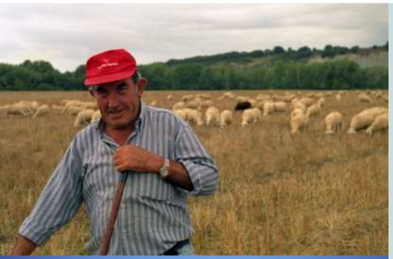

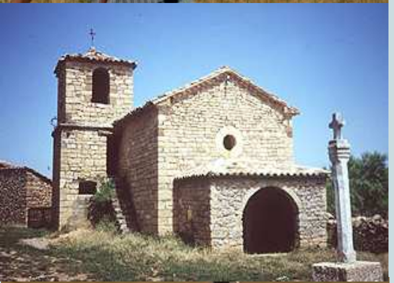

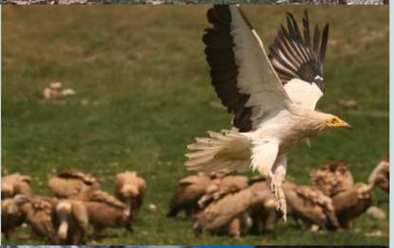

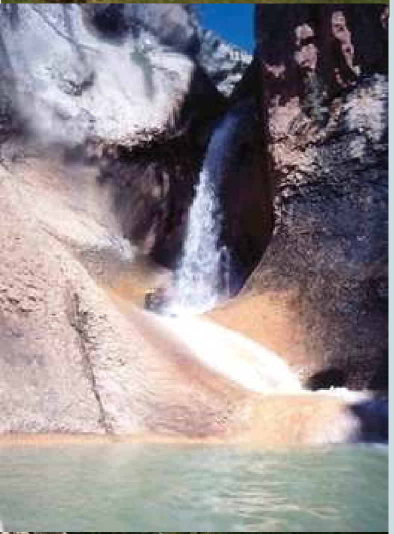

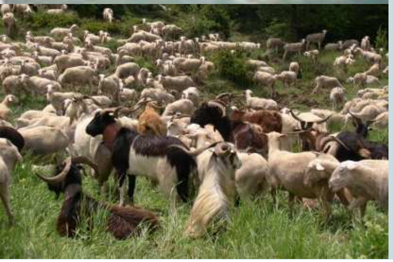

Parte 3. Ahora, algunas preguntas sobre usted...

1. Año de nacimiento ☐

2. Género   Mujer ○   Hombre ○

3. ¿Cuántas personas viven en su casa incluyéndole a usted? ☐

4. Lugar de residencia ☐

5. ¿Puede indicarnos su nivel de estudios?
- ○ - Educación primaria o básica
- ○ - Bachiller, formación profesional o similar
- ○ - Formación universitaria

6. ¿Están sus estudios o profesión relacionados con …?
- Medio ambiente   sí ○   no ○
- Agricultura y ganadería   sí ○   no ○

7. ¿Puede indicarnos el nivel de ingresos brutos de su familia? (todas las personas con ingresos que habitan en su hogar)
- ○ - menos de 700€ al mes
- ○ - entre 700 y 1500€ al mes
- ○ - entre 1500 y 3000€ al mes
- ○ - más de 3000€ al mes

8. ¿Puede indicarnos su profesión o dedicación actual?

☐

Ejemplo: funcionario, parado, ama de casa, estudiante, jubilado, etc.

9. ¿Tiene usted o alguien de su familia actividades agrarias?
○ sí   ○ no

10. ¿Es usted miembro de alguna cooperativa o asociación de consumidores?
○ sí   ○ no

11. ¿Es usted miembro de alguna organización de defensa de la naturaleza?
○ sí   ○ no

12. Si reside fuera del Parque de Guara, ¿cuántas veces lo ha visitado en los últimos 5 años?
- ○ - ninguna
- ○ - 1 o 2 veces
- ○ - de 3 a 5 veces
- ○ - más de 5 veces

13. Si ha visitado el parque alguna vez, ¿cuál fue el motivo principal? (elija una sola opción)
- ○ - caza
- ○ - senderismo
- ○ - barranquismo/ escalada
- ○ - observar la naturaleza
- ○ - otras (indicar) ☐

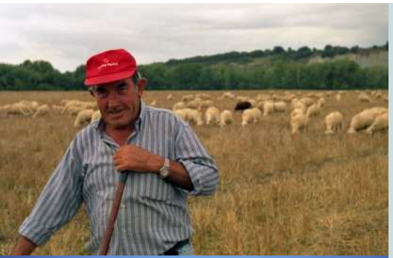
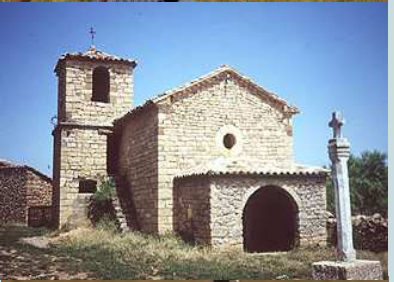
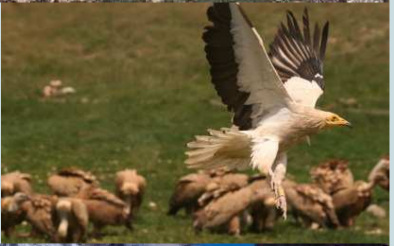
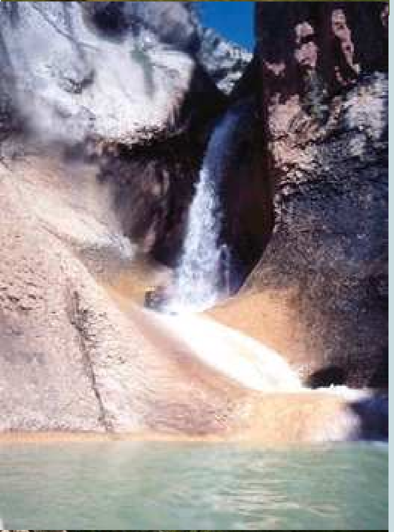
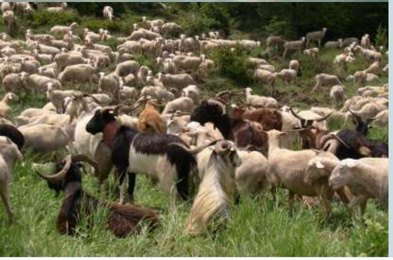

Si tiene alguna **sugerencia** adicional sobre cualquiera de los temas tratados en este cuestionario, por favor escríbala aquí:

Muchas gracias por su participación!

Si quiere contactar con nosotros, o tiene alguna duda o sugerencia, puede dirigirse a:
Raimon Ripoll Tel. 620342982 Correo electrónico: rripoll@aragon.es
Tamara Rodríguez Tel. 680796232 Correo electrónico: trodriguezo@cita-aragon.es

Grupo de Investigación en Sistemas Agro-silvo-pastorales Sostenibles
Centro de Investigación y Tecnología Agroalimentaria (CITA)
Avda. Montañana 930, 50059 Zaragoza
